# A triple fouling layers perspective on evaluation of membrane fouling under different scenarios of membrane bioreactor operation

**DOI:** 10.1186/2052-336X-12-91

**Published:** 2014-06-06

**Authors:** Mehdi Pourabdollah, Ayoob Torkian, Seyed Jamalodin Hashemian, Bita Bakhshi

**Affiliations:** 1 Faculty of Civil Engineering, Sharif University of Technology, Tehran, Iran; 2 Institute of Water and Energy, Sharif University of Technology, Tehran, Iran; 3 Department of Bacteriology, Faculty of Medical Sciences, Tarbiat Modares University, Tehran, Iran

**Keywords:** MBR, Operational scenarios, Triple fouling layers, Municipal wastewater, PCR, SMP, Protein, Carbohydrate

## Abstract

One of the main factors affecting membrane fouling in MBRs is operational conditions. In this study the influence of aeration rate, filtration mode, and SRT on hollow fiber membrane fouling was investigated using a triple fouling layers perspective. The sludge microbial population distribution was also determined by PCR method. Through various applied operational scenarios the optimal conditions were: aeration rate of 15 LPM; relaxation mode with 40s duration and 8 min. interval; and SRT of 30 days. The similarity between SMP variations in triple fouling layers with its corresponding hydraulic resistance confirmed the effect of SMP on membrane fouling. Among three fouling fractions, the upper (rinsed) layer found to have the most effect on membrane fouling which implies the critical role of aeration, but as for multilateral effects of aeration, the optimal aeration rate should be determined more precisely. Relaxation interval was more effective than its duration for fouling control. SRT variations in addition to influencing the amount of SMP, also affect on the structure of these material. At longer SRTs (20, 30 days) a greater percentage of SMP could penetrate into the membrane pores and for shorter SRTs they accumulate more on membrane surface. Results showed that there is a very good correlation between total hydraulic resistance (Log R) and protein to carbohydrate ratio at the rinsed layer (P1/C1). Considering significant effects of aeration and SRT conditions on this ratio (according to data), it is very determinative to apply the optimal aeration and SRT conditions.

## Introduction

Membrane bioreactors (MBRs) are widely used to treat municipal and industrial wastewaters [1]. Solids’ separation by membrane provides unique advantages over conventional activated sludge (CAS) systems including a smaller footprint, less sludge production and better effluent quality [2-4].

Membrane fouling remains a major operational issue leading to higher operational costs compared to current treatment technologies [1]. The main factors affecting membrane fouling include biomass characteristics (MLSS concentration, particle size distribution, concentrations of microbial products), operational conditions (aeration intensity, hydraulic retention time (HRT), solid retention time (SRT), operating flux, backwashing and chemical cleaning), and membrane physicochemical characteristics (pore size, surface characteristics, and chemical composition). [1,5-9].

Exocellular materials excreted from cells are considered important membrane foulants [10-13]. Extracellular polymeric substances (EPS) and soluble microbial products (SMP) contain carbohydrates and proteins, and humic substances, uronic acids and nucleic acids are present in smaller quantities [14]. According to study of Zhang et al., the initial stage of fouling includes passive adsorption of SMP and colloids on the membrane surface and initial pore blocking by feed particulates [15]. After this stage, the membrane surface is expected to be mostly covered by SMP, promoting attachment of biomass particulate and colloidal material during next stage [1]. The second stage consists of further pore blocking, biofilm growth due to accumulation of SMP and colloids, and cake formation by EPS bound within the biomaterials [15].

Previous studies have shown that humic and low molecular weight substances pass the membrane and therefore are not responsible for fouling, while polysaccharides (carbohydrates), proteins and organic colloids are retained almost completely [16]. Comprehensive review by Le-Clech et al. [9] indicates a direct relationship between soluble carbohydrates and fouling rate with a significant role played by the protein fraction. Effective operational parameters in decreasing importance include: aeration, sludge waste (which controls SRT), filtration mode, membrane cleaning, and imposed flux [9]. Aeration has three major roles: providing oxygen, maintaining the activated sludge in suspension and mitigating fouling [17] and SRT affects biological parameters like MLSS, SMP and eEPS concentrations [9].

**Figure 1:**
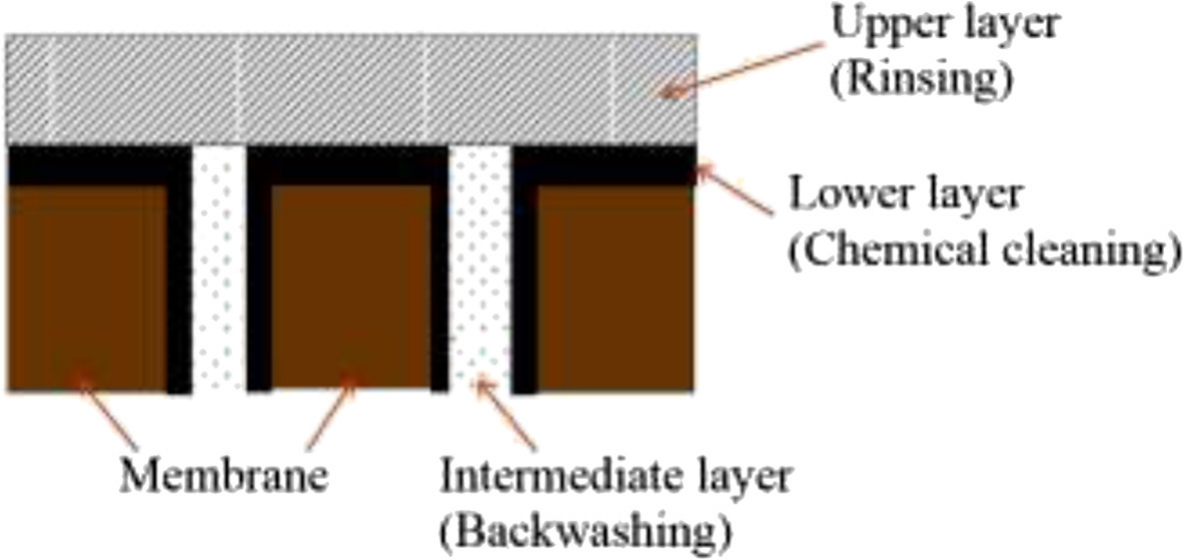
Fractionation of the fouling layer (and their method of recovery).

Also, few studies have investigated the membrane fouling with the approach of different fouling layers. As shown in Figure 1, fouling layers can be separated into three fractions, i.e. the upper (rinsed), intermediate (backwashed) and lower (desorbed) fouling layers [18-20]. Previous studies indicate the effect of different filtration modes (relaxation, backwashing, and mixed) on the membrane fouling [18-20].

According to the study of Wu et al. [18], the rinsed fraction contains sludge flocs and biopolymers, originating from the cake layer on the membrane surface. The backwashed fraction is mainly composed of materials which block the membrane pores, while the desorbed fraction represents irreversible fouling. The resistance of the rinsed fraction contributed more than that of the desorbed fraction and had significant correlations with transmembrane pressure (TMP). Wu et al. also indicated that characteristics of the foulants on membrane surface were similar to those in the mixed liquor in bioreactor [18].

Metzger et al. [19] mentioned that three different fractions have different compositions and consequences on the fouling resistance. The upper cake layer consists predominantly of loosely bound biomass flocs and attached SMP. The layer is characterized by a low specific biopolymer resistance and a high permeability. That is assumed to have a porous structure allowing water to permeate easily. An intermediate layer, is composed equally by SMP and biomass flocs or EPS clusters and features a higher specific biopolymer resistance than the upper layer. Soluble carbohydrates are accumulate in this layer. The layer has a denser matrix, is expected to fill up the pores and act like a gel-like layer between the lower membrane fouling layer and the upper cake layer. The desorbed layer is composed predominantly of SMP. This layer is intimately attached to the membrane and forms a total coverage of the surface and its pores. Compared to the two other layers, it contains a higher concentration of soluble proteins strongly bound to the membrane. It features a very dense structure and has a very low permeability, resulting in the highest specific biopolymer resistance [19].

Considering the results of these studies, the importance of measuring triple fouling layers is better understood. By this measurement, firstly the most effective layer on membrane fouling and thus its relevant control method (operational) could be determined. Also determination of effective components of SMP (protein, carbohydrate) at each layer is an important parameter for improving the interaction between these components and membrane surface, such as improving physical and chemical structure of the membrane and so on.

Differences seen between the results of these studies, could be due to different applied operational conditions (SRT, aeration rate …). So Further research is needed to highlight the impact of other operating conditions like aeration rate and SRT. In this study, the effects of different operating conditions (aeration rate, SRT, and filtration modes) on the membrane fouling were investigated by fractionating the triple fouling layers. The correlation analysis was used to find the more important parameter (protein, carbohydrate) affecting transmembrane pressure (TMP) variation, in each layer, and identify the effect of each layer on the total fouling resistance. The optimal operating condition is also determined.

## Materials and methods

### Experimental set up and tests

**Figure 2:**
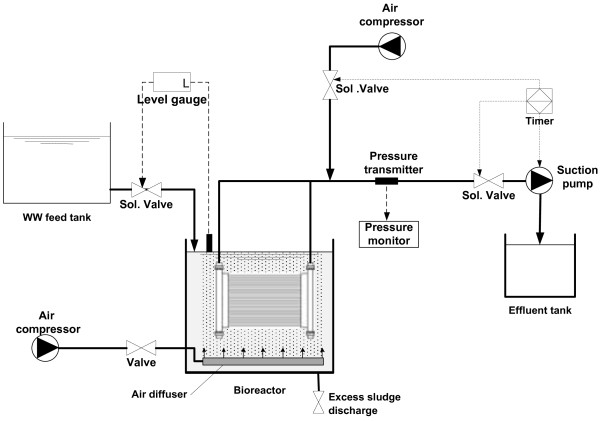
Schematic of the MBR rig.

The lab-scale aerobic MBR with a working volume of 26.4 L is shown in Figure 2. The membrane tank was equipped with a submerged hollow-fiber membrane module with a surface of 0.3 m^2^. The polyvinylidene difluoride (PVDF) membrane (Zenon, Canada) had a nominal pore size of 0.1 μm. The hydraulic retention time (HRT) of the aeration tank was 8 h and the flux through the membrane was 11 LMH (liter/m^2^/h). Aeration was provided with a blower through a porous air diffuser.

The bioreactor temperature was maintained at 29 ± 0.5°C during the experiments using an electric heater. TMP was continuously monitored by pressure transmitter.

The bioreactor was originally seeded with sludge collected from a local municipal wastewater treatment plant. The microbial population distribution of sludge was also determined by PCR (Polymerase chain reaction) method. In this regard, first a DNA extraction was done by QIAamp DNA Mini Kit (Qiagen, Hilden, Germany). Then the DNA was quantified using an ultra violet spectrophotometer. After executing PCR stages, the products was electrophoresed by agarose gel electrophoresis, stained with ethidium bromide, and documented using a gel documentation system. ABI 3730X capillary sequencer was used for DNA sequencing and finally the sequences were analysed by GeneRunner program. The distribution of bacteria’s identified were: *Enterobacter amnigenus* (83.5%), *Bacillus thuringiensis* (12.6%), *Aeromonas hydrophila* (3.9%).

The bioreactor was fed with synthetic wastewater [21]. Typical conditions consisted of influent COD of about 450 mg/L, TN of about 24.6 mg/L, and TP of about 5.1 mg/L (C:N:P ≈ 100:5:1).

Before running the experiments, the intrinsic resistance of the membrane (R_m_) was determined by clean water test. After an adaptation stage, each experiment was executed in a 24 h period. For each series of scenarios, the optimal operational parameters of the previous series were applied. The first series of scenarios (aeration) were executed under typical operating conditions (SRT = 30 days, backwash duration and interval = 40 s and 8 min.).

During the filtration period, TMP was monitored hourly and total hydraulic resistance after 24 h was calculated based on Darcy’s law (Eq. 1).

(1) $$R_{\text{total}}=\frac{\text{TMP}}{\mu J}$$

Where J is the flux and R_total_ is the resistance after 24 h. TMP and μ are the trans-membrane pressure and the dynamic viscosity of permeate (water), respectively.

After each filtration period of 24 h, the fouled membrane was cleaned following a three-step protocol [18]: (1) rinsed with 200 mL distilled water (2) backwashed with 1000 mL distilled water (3) desorbed in 1000 mL NaOH solution (pH 12) for 24 h. By applying this specific protocol, the fouling layer could be separated into three fractions, i.e. rinsed, backwashed and desorbed. After each step, a suction test was applied again to measure the resistance of each fraction and the three cleaning solutions were analyzed in terms of carbohydrate and protein concentration (SMP calculated as the sum of carbohydrate and protein concentrations).

Protein concentration was measured by the Lowry method, modified by Peterson using bovine serum albumin as standard. Samples were measured at 720 nm [22,23]. Polysaccharide concentration was measured by the phenol–sulfuric acid methods with glucose used as standard. Samples were analyzed at 490 nm [24].

### Scenarios

Three different conditions for aeration rate (A1 = 0.5, A2 = 1.2, and A3 = 4 m^3^/m^2^.h or A1 = 2.5, A2 = 6, and A3 = 15 lit/min (LPM)) were selected based on typical values mentioned in a literature review on aeration of MBRs [25]. After 2 days of adaptation for each scenario, the 24 h test was done.

After aeration scenarios, two series of filtration scenarios (backwashing and relaxation) and a continuous mode were applied to the MBR (Table 1). Duration and interval values were selected based on previous studies such as the study of Wu. et al. [18]. The optimal aeration rate determined in the previous experiments, was used in filtration scenarios.

**Table 1:** Conditions applied in the filtration experiments

**Scenario**		**Duration (s)**	**Interval (s)**
Continuous	C1	-	-
Relaxation	Ra	20	480
	Rb	20	240
	Rc	40	480
Backwash	B1	40	240
	B2	20	240
	B3	40	480
	B4	20	480

After completing these experiments and selecting the optimal filtration mode, the SRT scenarios were initiated. Three different solid retention times including S1 = 10 days, S2 = 20 days, and S3 = 30 days were applied, based on typical SRT values in previous studies [4,26,27]. The optimal aeration rate and filtration mode determined in the prior experiments were used.

## Results and discussions

**Figure 3:**
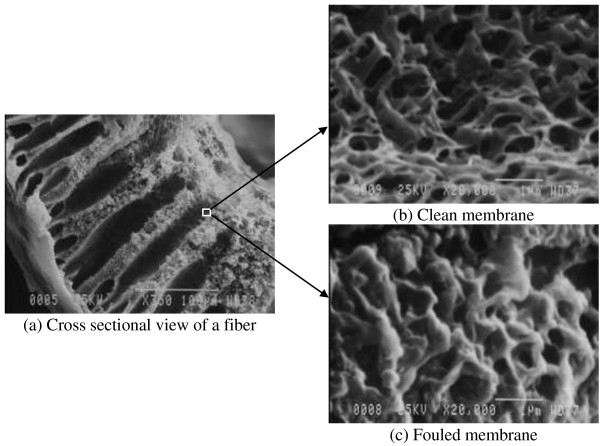
SEM images of clean and fouled HF membrane pores (intermediate layer of fouling): (a) cross sectional view of a fiber; (b) clean membrane; (c) fouled membrane.

An example of SMP accumulation in the membrane pores (intermediate fouling layer) at the end of a 24 h operational scenario is shown in the SEM images of a clean and fouled membrane fiber (Figure 3). As seen, a portion of the pores volume (in comparison with clean membrane) is filled by the fouling material which appeared to be like a dense gel material.

### Aeration scenarios

**Figure 4:**
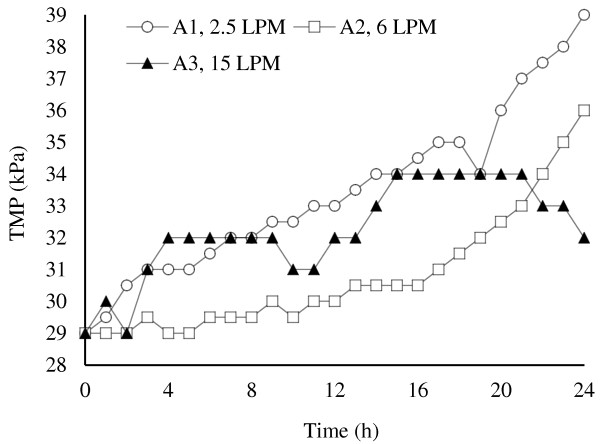
Temporal profile of TMP for different aeration conditions.

Temporal TMP profiles of aeration scenarios shown in Figure 4 indicate a clear difference between their fouling trends.

**Figure 5:**
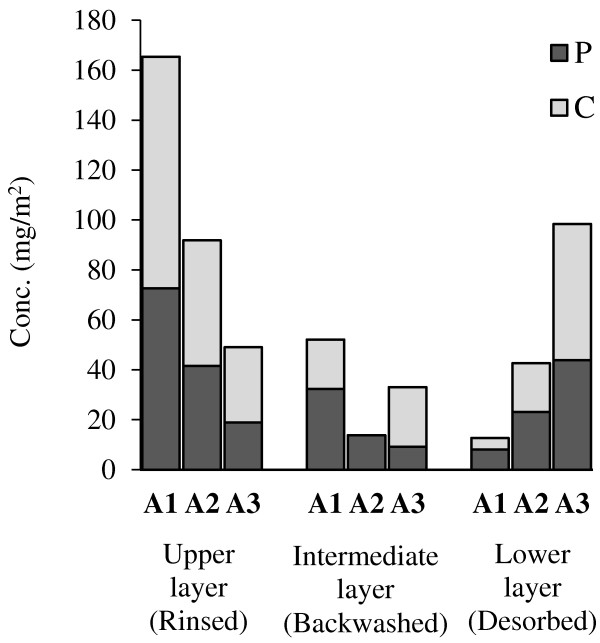
SMP content (sum of P & C) in different fouling fractions at different aeration runs.

All three scenarios had a nearly increasing fouling behavior, but A3 showed a smoother profile with a decreasing final TMP, so that the TMP at the end of this run was clearly lower than the others. Also COD removal in this scenario was higher than the others (A1: 68%, A2: 91%, A3: 93%). Therefore A3 (15 LPM) was selected as the optimal aeration condition for the subsequent experiments. This indicate that in the range of applied aeration conditions (up to 15 LPM), higher aeration rates result in better fouling control.SMP contents (sum of protein “P” and carbohydrate “C”) in the fouling fractions are shown in Figure 5.

**Figure 6:**
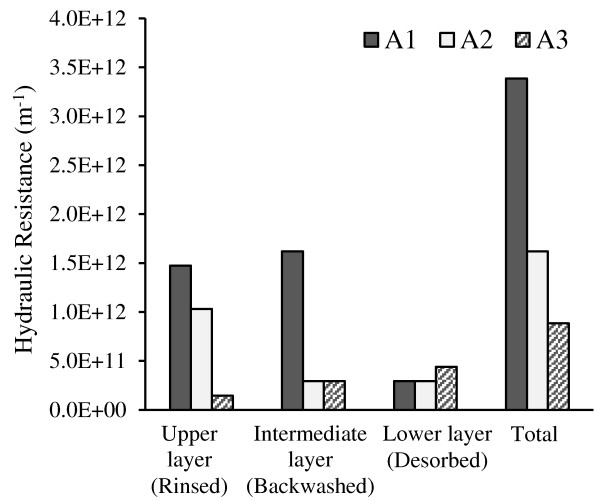
Resistance for the different fractions under the different aeration conditions.

As observed in A1 and A2 runs, the rinsed fractions contained more SMP than the other fractions. But in A3, the desorbed fraction had more SMP content than the other fractions. This situation implies that by increasing aeration rate from A1 to A3, the SMP content in the rinsed layer decreases due to higher shear stress on the membrane surface, and this leads to lower SMP content in the backwashed layer. But increasing aeration rate may also damage the floc structure, reduce their size, and release SMP in the bioreactor [28,29]. The released SMP cannot accumulate more at the rinsed layer, because of high shear stress. But it causes higher SMP concentrations in the backwashed and desorbed layer. Increase of SMP concentration in Figure 5 for desorbed layer (from A1 to A3) and backwashed layer (from A2 to A3), clearly imply this phenomenon.As shown in Figure 6, increasing aeration rate from A1 to A3, resulted in a considerable decrease of total hydraulic resistance, so the run A3 featured the lowest final resistance. The resistance decrease is also observed in the rinsed and backwashed layers (from A1 to A3), but no significant effect due to aeration rate is detected on the resistance of desorbed layer.

### Filtration scenarios

**Figure 7:**
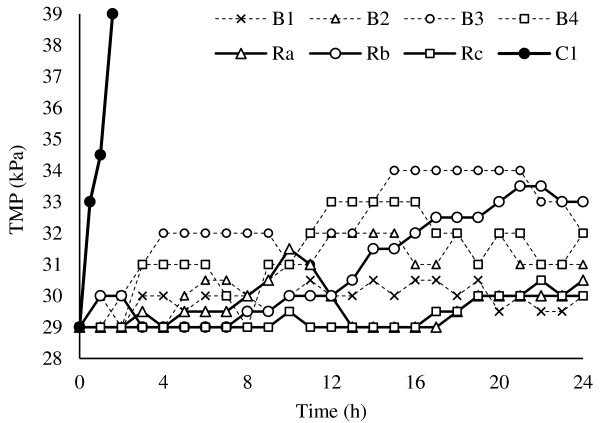
TMP vs time for different filtration scenarios.

Figure 7 shows all the filtration scenarios TMP temporal profiles. As observed, the continuous mode (C1) exhibited a very rapid rise of TMP in the first hours of run, due to severe blocking of membrane pores. So the use of a physical cleaning mode is inevitable.Comparing temporal TMP profiles in Figures 3 and 6, indicates that except for B3 (which equaled to A3) other backwash and relaxation scenarios had better performance on fouling control than aeration scenarios. Also among filtration experiments, relaxation modes showed lower TMP profiles than backwash modes (Figure 7).

In addition, backwash scenarios with shorter intervals (240 s) showed better performance in fouling control than longer intervals (480 s). But in relaxation scenarios, longer intervals (480 s) exhibited a more efficient effect on TMP control than the shorter interval (240 s).

**Figure 8:**
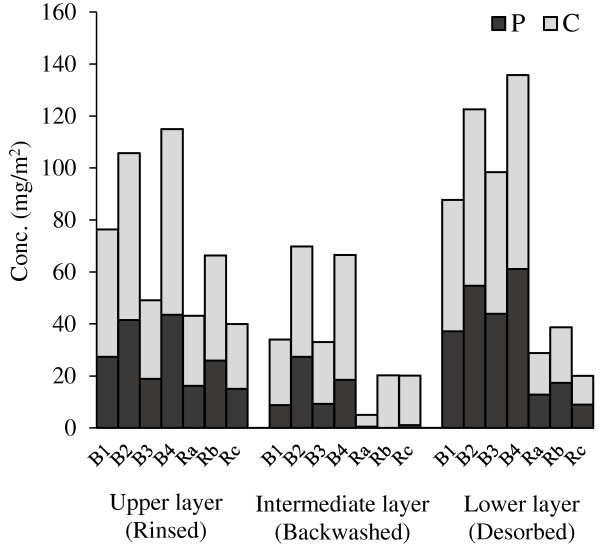
SMP content (sum of P & C) in different fouling fractions at different filtration runs.

**Figure 9:**
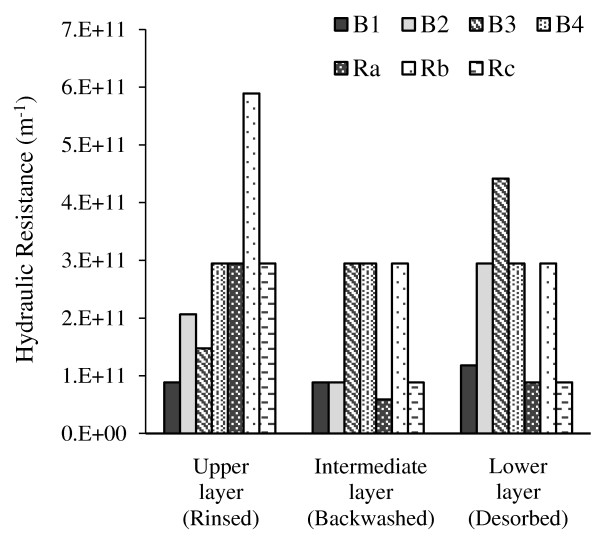
Resistance for the different fractions under the different filtration conditions.

By comparing the different relaxation durations (20, 40 s), it could be found that duration did not affect TMP as much as interval, which is similar with the results of Jinling Wu et al. study [18].As seen in Figure 7, the run B1 had a final TMP equal to run Rc, but the overall TMP profile of Rc is lower than B1. Also, Rc showed a lower TMP profile than Ra. So, it can be concluded that Rc (Relaxation with 40 s duration and 8 min. interval) is the optimal filtration scenario.According to Figure 8, the SMP content of fouling fractions observed in relaxation modes were lower than backwash modes (especially in backwashed and desorbed layers). Comparing with the optimal aeration scenario (A3) in which B3 was used as filtration mode, other backwash runs (B1, B2 and B4) showed an inefficient performance on controlling SMP accumulation. But all relaxation runs had acceptable efficiencies in comparison with A3 (or B3).Compared with backwashing, relaxation had a weaker effect on rinsed layer, but it exhibited a more positive effect on the other fouling layers, except for run Rb (Figure 9). The run Rb resulted in more than twice hydraulic resistance in comparison with runs Ra and Rc, in all layers. This confirms the importance of interval than duration in relaxation modes.

### SRT scenarios

**Figure 10:**
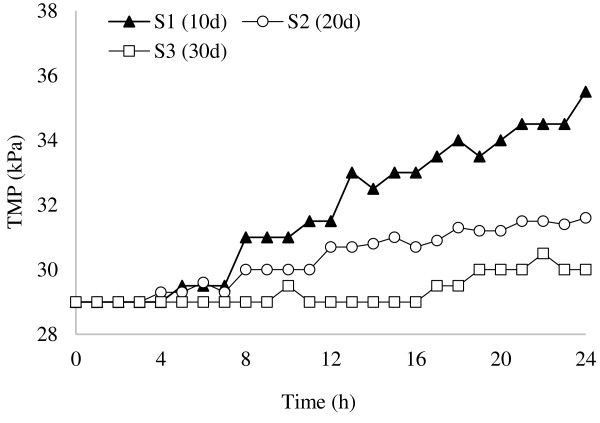
TMP vs time for different SRT scenarios.

The TMP profiles of SRT scenarios are shown in Figure 10. As shown, S3 had a significant lower TMP profile than the others, so clearly it is selected as the optimal SRT scenario. S1 showed the highest TMP profile and S2 had the intermediate profile.

**Figure 11:**
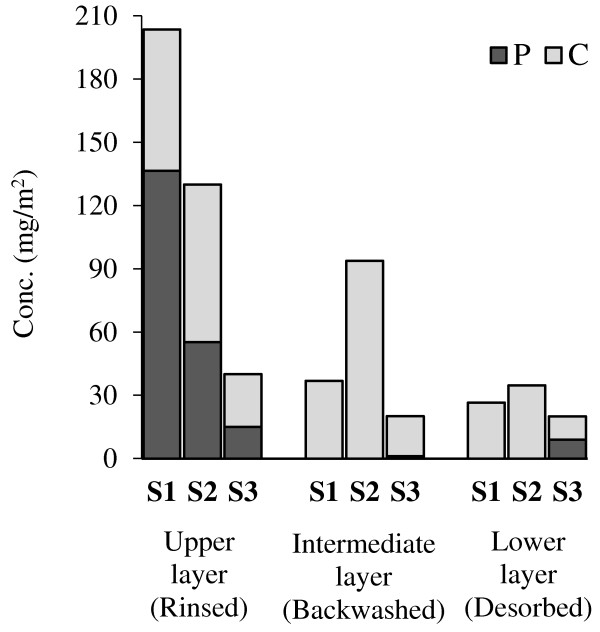
SMP content (sum of P & C) in different fouling fractions at different SRT runs.

**Figure 12:**
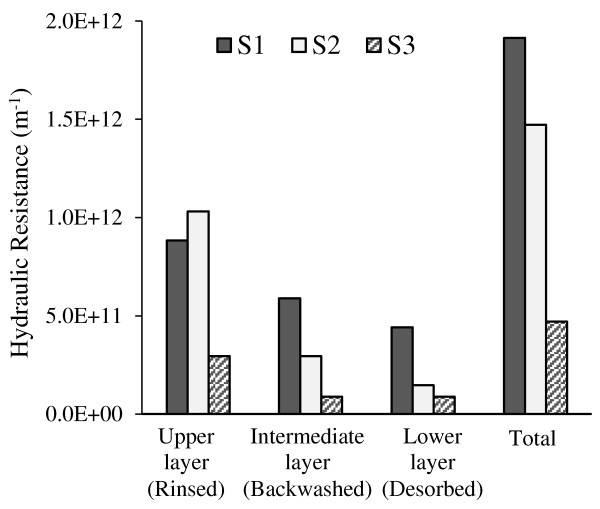
Resistance for the different fractions under the different SRT conditions.

SMP contents distribution in the fouling fractions (Figure 11) indicated that in the runs S1 and S2 (SRT = 10, 20 days), considerable high concentrations of SMP produced than in S3 (SRT = 30 days). Higher SMP content of S3 is a result of the lower sludge bioactivity at the longer SRT (30 d). OUYANG Ke et al. (2009) showed this phenomenon by quantifying the sludge bioactivity in the MBR using the fluorescence in situ hybridization (FISH) method [30]. Also, in S1 most of the SMP content accumulated at the rinsed layer, but for S2 and S3 a greater percentage of SMP penetrated into the membrane pores. This implies that fouling (SMP) structure at SRT of 30 days is different from those of 10 and 20 days.As seen in Figure 11, all the protein content of SMP in the runs S1 and S2 was accumulated in rinsed layer. This could be a reason for the different fouling structure of rinsed layer at SRT of 10 and 20 days (which mentioned in previous paragraph).In SRT scenarios, S1 showed the highest total hydraulic resistance at the end of 24 h period and S3 had the lowest total resistance (Figure 12). Also, in all fouling fractions, by increasing SRT the hydraulic resistance at each layer decreased (ignoring a little difference at rinsed layer).

### Relationships

**Figure 13:**
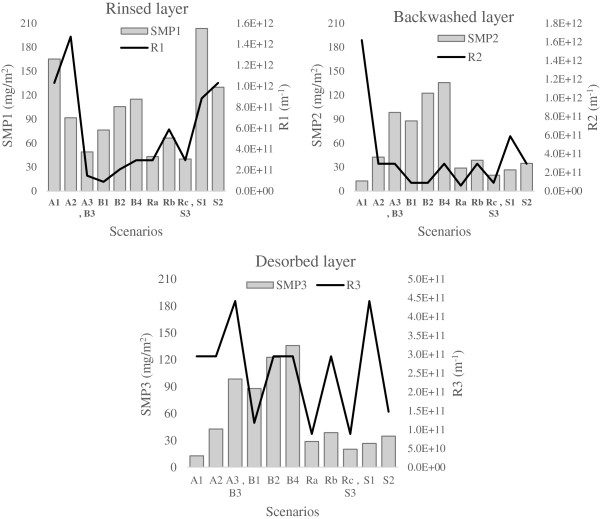
Variation of SMP and hydraulic resistance in all scenarios at different fouling layers.

Comparing hydraulic resistance (R) and SMP variations of all scenarios at each fouling layer (Figure 13), a similarity is observed between the states of variations of these two parameters, except for scenario A1 toward A2 and S1 toward S2. Because of multiple roles of aeration used in MBR system on fouling, transition from A1 to A2 caused some different effects that finally resulted in such behavior. Also structural change of SMP due to SRT variation (discussed before) caused the difference between SMP and R variation from S1 to S2.

But totally, the similarity observed between SMP and R variations, confirms the relationship between fouling and SMP content in MBR system (according to previous studies).

Also a correlation matrix of some parameters including: P, C, SMP, R, and some of their combinations considering each fouling layer, has been calculated based on Pearson coefficient (r). The significant correlations are separated and gathered in Table 2, with emphasis on hydraulic resistance (R) as the fouling indicator. No significant correlation was found between other parameters.

**Table 2:** Significant correlations (Pearson coef.) between some parameters

**r**	**Log R1**	**Log R2**	**Log R**
P1	-	0.747 (good)	0.737 (good)
C1	-	0.662 (good)	-
SMP1	-	0.706 (good)	0.663 (good)
P1/C1	0.885 (very good)	0.681 (good)	0.899 (very good)
Log R1	-	-	0.859 (very good)
Log R2	-	-	0.898 (very good)
Log R	0.859 (very good)	0.898 (very good)	-

As seen, total hydraulic resistance (Log R) showed the strongest correlation (r = 0.899) with protein to carbohydrate ratio at the rinsed layer (P1/C1). Comparing Pn/Cn ratios of different operational scenarios indicated that filtration scenarios had no significant effect on this ratio variations, but different aeration and SRT scenarios could alter this ratio significantly. In this regard, selecting optimal aeration and SRT conditions will be very important.

There was also a good correlation between Log R and SMP1 at the rinsed layer and especially its protein content. These findings (together with above paragraph) imply that the rinsed layer plays a major role in the membrane fouling and SMP content and its components (especially protein) at this layer should be considered as the main parameters for fouling control.

Furthermore it observed that among three fouling fractions, the rinsed and backwashed layers (Log R1 and R2) had very good correlations to the total hydraulic resistance (Log R), but desorbed layer (R3) did not show such relationship. So fouling control actions should be concentrated enough on these two layers.

## Conclusions

In this study, the effects of different conditions of aeration rate, filtration mode, and SRT were assessed on fouling mitigation in MBR system. Important conclusions could be drawn:

• Optimal operational conditions found among executed scenarios were: scenario A3 as aeration rate (15 LPM), scenario Rc as filtration mode (Relaxation with 40 s duration and 8 min. interval), and scenario S3 as SRT (30 days).

• Comparing SMP variations with hydraulic resistance variations in different operational scenarios (especially in Figure 13), totally showed a similarity between these two variations, which confirms the relationship of membrane fouling with SMP in MBR system (according to previous studies).

• The rinsed layer found to be the most effective fraction of membrane fouling considering SMP and hydraulic resistance graphs (Figures 4, 5, 7, 8, 10, and 11). Also the good correlation between SMP content (especially protein) in this fraction confirms its major role in membrane fouling. In this regard, the aeration should be considered and applied as a very important practice with the ability of controlling this fouling layer.

• Aeration exhibits multiple effects with different aspects on membrane fouling, so at each aeration rate some specific effects were dominant and hence its corresponding fouling behavior was not uniform for all of the aeration rates. This situation is clearly observed in transition of aeration rate from A1 to A2 scenario (comparing SMP and R variations). So the optimal aeration rate should be determined more precisely.

• Relaxation in comparison with air backwashing showed a more positive effect on fouling control, and also its interval was more important than its duration for fouling control.

• SRT variations in addition to influencing on the amount of produced exocellular materials, also affect on the structure of these material, so that at longer SRTs (20, 30 days) a greater percentage of SMP could penetrate into the membrane pores and for shorter SRTs they accumulate more on membrane surface. These effects thought to be due to the amount of protein production at each SRT (section “SRT scenarios”).

• Results showed that there is a very good correlation between total hydraulic resistance (Log R) and protein to carbohydrate ratio at the rinsed layer (P1/C1). Considering significant effects of aeration and SRT conditions on this ratio, it will be very determinative to apply the optimal aeration and SRT conditions.

## Competing interests

The authors declare that they have no competing interests.

## Authors’ contributions

MP has designed, constructed and operated the pilot unit, analyzed the data and prepared the manuscript. AT supervised the study and commented on the first draft. SJH was advisor of the study. BB contributed in microbial population identification. All authors read and approved the final manuscript.
